# Editorial: Non-Coding RNA Mediated Post-Transcriptional Regulation in Human Diseases

**DOI:** 10.3389/fgene.2022.901664

**Published:** 2022-05-09

**Authors:** Santosh Kumar, Shaveta Kanoria

**Affiliations:** ^1^ Life Science Department, National Institute of Technology, Rourkela, India; ^2^ Wadsworth Center, Center for Medical Science, New York State Department of Health, Albany, NY, United States

**Keywords:** non-coding RNA, lncRNA, circRNA, post-transcriptional regulation, miRNA

Recent advances in the technology and analysis aspect of RNA sequencing have unraveled many non-coding RNAs. These non-coding RNAs regulate various post-transcriptional processes and cellular functions under normal and diseased conditions. In this research topic, the published articles cover a vast diversity of non-coding RNA types, including long non-coding RNA (lncRNA), circular RNA (circRNA), transfer RNA (tRNA), and microRNA (miRNA). The objective of this topic is to highlight the intricate relationship of ncRNA with post-transcriptional processes and their role in human diseases. A total of five research articles and four reviews are published. These research articles and reviews focusing on the objective of this topic explore the role of ncRNAs in a range of physiological processes and human diseases, including colorectal cancer, lung function and diseases, pulmonary hypertension, cystic fibrosis, adipogenesis, and reproduction.

The article by Fei Yao et al. investigated the circular RNA profiles and colorectal cancer (CRC) chemoresistant cell lines. They studied the differential expression of circRNA and analyzed their role in the chemoresistance of CRC. In another research, Pengpeng Zhang et al. studied the expression of circular RNA using RNA sequencing in brown adipogenesis at various differentiation stages. They utilized an array of computational tools to understand and predict the potential role of circRNA in brown adipogenesis. The review article by Soni and Biswas highlighted the role of lncRNA and miRNA in the post-transcriptional regulation of several lung diseases, including asthma, chronic obstructive pulmonary disease, cystic fibrosis, and idiopathic pulmonary fibrosis. Another review article by Chaofan He et al. explored the role of non-coding RNA in reproductive processes and reproductive diseases. They described the role of miRNA, lncRNA, and PIWI-interacting RNA in spermatogenesis and follicular development. In addition, they also discussed the role of non-coding RNA in male and female reproductive diseases.

In a pilot study of X-linked microRNA expression, McKiernan et al. studied the top seven X-linked microRNAs, namely, miR-224-5p, miR-452-5p, miR-450-5p, miR-542-3p, miR-450a-5p, miR-424-5p, and miR-545-5p, which are significantly upregulated in cystic fibrosis versus non-cystic fibrosis monocytes correlating with lung function. Further, they found miR-224-5p to be correlating with lung function in cystic fibrosis, whereas the miR-545-5p and miR-224-5p levels correlate with exacerbation rate. Zhou et al. demonstrated the potentially extensive involvement of tRNA-derived fragments (tRFs) in cancers and provided a reasonable list of cancer-associated tRFs for further investigations. Another review article by Xia et al. described the role of lncRNAs in renal fibrosis (RF). They evaluated the recent publications on lncRNAs in RF and the potential applications of lncRNAs as diagnostic and prognostic biomarkers in RF. They also proposed potential therapeutic targets for treating RF-associated diseases and subsequent CKD. The article by Liu et al. indicated that the lncRNA KCNQ1OT1 ceRNA network could be involved in regulating the CRC tumor microenvironment. Interestingly, they found that lncRNA KCNQ1OT1 was significantly upregulated in CRC tissues and inversely associated with the survival of patients, indicating KCNQ1OT1 as a possible functional contributor and therapeutic target for CRC. The review article by Zhang et al. elucidated the involvement of non-coding RNA networks in pulmonary hypertension. The authors constructed ncRNA networks by assembling ncRNAs and their interacting RNAs or genes, providing a better understanding of the roles of ncRNAs in pulmonary hypertension and potential clinical applications of the ncRNAs in pulmonary hypertension.

The Research Topic of articles in this topic advances our understanding of the role of ncRNA in various post-transcriptional processes and human health and diseases.

